# Is the presence of foraminal stenosis associated with outcome in lumbar spinal stenosis patients treated with posterior microsurgical decompression

**DOI:** 10.1007/s00701-023-05693-5

**Published:** 2023-07-05

**Authors:** Jørn Aaen, Hasan Banitalebi, Ivar Magne Austevoll, Christian Hellum, Kjersti Storheim, Tor Åge Myklebust, Masoud Anvar, Clemens Weber, Tore Solberg, Oliver Grundnes, Helena Brisby, Kari Indrekvam, Erland Hermansen

**Affiliations:** 1 Department of Orthopedic Surgery, Ålesund Hospital, Møre and Romsdal Hospital Trust, Ålesund, Norway; 2 Department of Circulation and Medical Imaging, Faculty of Medicine and Health Sciences, Norwegian University of Science and Technology, Trondheim, Norway; 3 Department of Diagnostic Imaging, Akershus University Hospital, Nordbyhagen, Norway; 4 Institute of Clinical Medicine, University of Oslo, Oslo, Norway; 5 Kysthospitalet in Hagevik, Orthopedic Clinic, Haukeland University Hospital, Bergen, Norway; 6 Department of Clinical Medicine, University of Bergen, Bergen, Norway; 7 Division of Orthopedic Surgery, Oslo University Hospital Ullevaal, Oslo, Norway; 8 Communication and Research Unit for Musculoskeletal Health (FORMI), Oslo University Hospital, Oslo, Norway; 9 Department of Research and Innovation, Møre and Romsdal Hospital Trust, Ålesund, Norway; 10 Unilabs Radiology, Oslo, Norway; 11 Department of Neurosurgery, Stavanger University Hospital, Stavanger, Norway; 12 Dept. of Quality and Health Technology, University of Stavanger, Stavanger, Norway; 13 Department of Neurosurgery and the Norwegian Registry for Spine Surgery (NORspine), University Hospital of Northern Norway, Tromsø, Norway; 14 Institute of Clinical Medicine, The Arctic University of Norway, Tromsø, Norway; 15 Department of Orthopedics, Akershus University Hospital, Nordbyhagen, Norway; 16 Dept of Orthopaedics, Sahlgrenska University Hospital, Gothenburg, Sweden; 17 Dept. of Orthopaedics, Institute for Clinical Sciences, Sahlgrenska Academy, University of Gothenburg, Gothenburg, Sweden

**Keywords:** LSS, Outcome, Foraminal stenosis, Disk degeneration, NORDSTEN

## Abstract

**Background:**

We aim to investigate associations between preoperative radiological findings of lumbar foraminal stenosis with clinical outcomes after posterior microsurgical decompression in patients with predominantly central lumbar spinal stenosis (LSS).

**Methods:**

The study was an additional analysis in the NORDSTEN Spinal Stenosis Trial. In total, 230 men and 207 women (mean age 66.8 (SD 8.3)) were included. All patients underwent an MRI including T1- and T2-weighted sequences. Grade of foraminal stenosis was dichotomized into none to moderate (0–1) and severe (2–3) category using Lee’s classification system. The Oswestry Disability Index (ODI), Zurich Claudication Questionnaire (ZCQ), and numeric rating scale (NRS) for back and leg pain were collected at baseline and at 2-year follow-up. Primary outcome was a reduction of 30% or more on the ODI score. Secondary outcomes included the mean improvement on the ODI, ZCQ, and NRS scores. We performed multivariable regression analyses with the radiological variates foraminal stenosis, Pfirrmann grade, Schizas score, dural sac cross-sectional area, and the possible plausible confounders: patients’ gender, age, smoking status, and BMI.

**Results:**

The cohort of 437 patients presented a high degree of degenerative changes at baseline. Of 414 patients with adequate imaging of potential foraminal stenosis, 402 were labeled in the none to moderate category and 12 in the severe category. Of the patients with none to moderate foraminal stenosis, 71% achieved at least 30% improvement in ODI. Among the patients with severe foraminal stenosis, 36% achieved at least 30% improvement in ODI. A significant association between severe foraminal stenosis and less chance of reaching the target of 30% improvement in the ODI score after surgery was detected: OR 0.22 (95% CI 0.06, 0.83), *p*=0.03. When investigating outcome as continuous variables, a similar association between severe foraminal stenosis and less improved ODI with a mean difference of 9.28 points (95%CI 0.47, 18.09; *p*=0.04) was found. Significant association between severe foraminal stenosis and less improved NRS pain in the lumbar region was also detected with a mean difference of 1.89 (95% CI 0.30, 3.49; *p*=0.02). No significant association was suggested between severe foraminal stenosis and ZCQ or NRS leg pain.

**Conclusion:**

In patients operated with posterior microsurgical decompression for LSS, a preoperative severe lumbar foraminal stenosis was associated with higher proportion of patients with less than 30% improvement in ODI.

**Trial registration:**

The study is registered at ClinicalTrials.gov (22.11.2013) under the identifier NCT02007083.

## Introduction

Degenerative changes of the lumbar spine can lead to the clinical condition known as lumbar spinal stenosis (LSS). The stenosis can be predominantly central, lateral, or foraminal. A combination of these findings was first described by Henk Verbiest [32]. LSS is characterized by clinical symptoms of back and leg pain, neurogenic claudication, and radiological images of the spinal canal indicating reduced space for neural structures in the spinal canal and the neuroforamina. After surgery, 60–80% of the patients report substantial improvement measured with different patient-reported outcome measurements (PROMs) [2, 12, 17, 21]. Open laminectomy and minimal invasive decompression procedures are utilized to treat LSS with equal outcome [26]. Several minimally invasive surgical procedures are commonly used with similar results regarding success and complication rates [15]. The main aim of the surgical interventions treating LSS is to decompress the neural structures within the spinal canal and the neural foramina. The pathophysiology of LSS, however, consists of several processes such as bulging of the disk, thickening of the flavum ligament, and growth of the facet joint leading to a narrowing of the spinal canal. With degeneration of the intervertebral disk and degenerative hypertrophy of the facet joint, the volume of the neural foramina diminishes, resulting in less space available for the spinal nerve root exiting the spinal canal through each foramen [29]. The observation of foraminal stenosis on MRI is a pathology potentially contributing to the LSS syndrome [27, 33]. While the neural structures within the spinal canal mostly are relived after posterior microsurgical decompression, restoring space in the foramen may require more complex surgery including resection of the facet joint, interbody fusion surgery, or far lateral approaches. Earlier studies have suggested that persisting foraminal stenosis can be linked to unfavorable outcomes after lumbar decompressive surgery [6]. Thus, the aim for this study was to investigate if foraminal stenosis seen on baseline MRI was associated with change in patient-reported outcome after microsurgical posterior decompression among patients with predominantly central lumbar spinal stenosis symptoms.

## Methods

The NORwegian Degenerative spondylolisthesis and spinal STENosis (NORDSTEN) study has three arms, two randomized controlled trials and one observational cohort with 988 included patients. NORDSTEN is a multicenter study done in collaboration with 18 Norwegian hospitals that routinely perform spine surgery and are evaluating clinical and radiological findings in patients with LSS. The patients in the present analysis are from the randomized NORDSTEN Spinal Stenosis Trial (NORDSTEN-SST), and consequently, no patients with degenerative spondylolisthesis are included [14].

### Inclusion process and patient recruitment

**Fig. 1:**
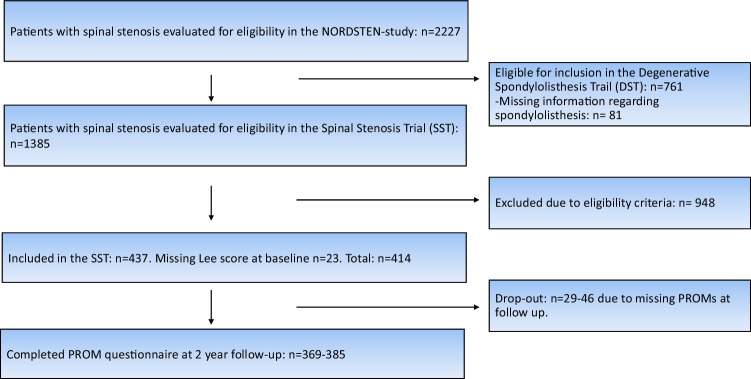
Flow chart of the NORDSTEN-SST investigating influence of foraminal stenosis. DST, Degenerative Spondylolisthesis Trial; SST, Spinal Stenosis Trial

The included patients had MRI findings and clinical symptoms consistent with LSS, i.e., symptoms of predominantly central stenosis but not specific nerve root pain (radiculopathy). Of 2227 patients screened for eligibility at orthopedic and neurosurgical departments at 16 Norwegian public hospitals, 437 patients fulfilled the eligibility criteria and were included in NORDSTEN-SST (Fig. 1). The enrolment of patients took place between February 2014 and October 2018. The patients were randomized to surgical treatment with one of three commonly used surgical techniques. All three techniques are labeled as minimal invasive and resulted in similar success rates in the present cohort [15]. None of the techniques included foraminotomy, lateral approaches, or interbody devices. The included patients answered questionnaires preoperatively and at a 2-year follow-up. Eligibility criteria are given in Table 1.

**Table 1:** Inclusion and exclusion criteria for the Spinal Stenosis Trial (SST) in the NORDSTEN study

Inclusion criteria	
Presence of clinical symptoms of spinal stenosis, such as neurogenic claudication or pain radiating bilaterally to the lower limbs	
Non-response to at least 3 months of non-surgical treatment	
Radiological findings corresponding to the clinical symptoms of LSS. Central-stenosis or lateral recess-stenosis	
Able to give informed consent and to answer the questionnaires	
Over 18 years of age	
Able to understand Norwegian, both spoken and written	
Exclusion criteria	
Degenerative lumbar spondylolisthesis, with a slip ≥ 3 mm verified on standing plain x-rays in lateral view	
Not willing to give written consent	
Previous surgery at the level of stenosis	
Fracture or former fusion in the thoraco-lumbar region	
Cauda equina syndrome (bowel or bladder dysfunction) or fixed complete motor deficit	
ASA-classified 4 or 5	
Over 80 years of age	
Presence of a lumbosacral scoliosis of more than 20 degrees, verified on AP view	
Presence of distinct symptoms in one or both legs, due to other diseases, e.g., polyneuropathy, vascular claudication, or osteoarthritis	
LSS at 4 or more levels	
Unable to comply fully with the protocol, including treatment, follow-up, or study procedures (psychosocially, mentally, or physically)	
The patient is participating in another clinical trial that may interfere with this trial	

### Magnetic resonance imaging

All participants underwent MRI of the lumbar spine within 6 months before surgery (1.5 or 3 Tesla). The MRI protocol dictated sagittal T1- and axial and sagittal T2-weighted images with repetition time (TR)/echo time (TE) 1500–6548/82–126 ms for T2-weighted images and 400–826/8–14 ms for T1-weighted images, slice thickness: 3–5 mm, and FOV: 160–350 mm. The MRI examinations were anonymized and the PACS IDS7 (SECTRA) was used for the assessment of morphological changes.

### Radiological variables

The inter- and intra-observer agreement analysis regarding the preoperative MRI examinations is reported in a previous study [5]. The index level was defined as the narrowest lumbar level measured by dural sac cross-sectional area (DSCA) [7]. At the index level, the grade of foraminal stenosis was classified using sagittal T1-weighted images according to the classification system proposed by Lee et al. [24]. The grading consists of scores 0–3.

- 0 = fat surrounding nerve in all directions
- 1 = fat lost only horizontally or vertically
- 2 = fat lost both horizontally or vertically
- 3 = fat lost both horizontally or vertically with morphological changes the nerve root

We dichotomized the radiological scores into none to moderate changes (Lee 0–1) or severe changes (Lee 2–3) in accordance with earlier publications [5]. The side with more severe changes (right/left) was used in the analysis.

A decreased disk height is likely associated with the development of foraminal stenosis [18]; therefore, disk degeneration (Pfirrmann grade) was included in the statistical model as a covariate.

Previous studies have indicated an association between Schizas score and DSCA and outcome. Therefore, these parameters were included in the analysis as covariates, although such associations are not suggested generally [7, 25].

A thorough discussion of the dichotomization of investigated covariates (Schizas, DSCA, and Pfirrmann) is presented in earlier publications [1, 7]. The radiological covariates were dichotomized into moderate or severe changes

### Outcome measures

The following patient-reported outcome measurements (PROMs) were collected at preoperatively and at 2-year follow-up after surgery: the Norwegian validated versions of the Oswestry Disability Index (ODI), the Zurich Claudication Questionnaire (ZCQ), and the numeric rating scale (NRS) for leg and back pain.

The ODI is a low back pain–specific questionnaire consisting of ten questions about pain-related disability. The ODI score ranges from 0 (no disability) to 100 (most severe disability) [9, 11].

The ZCQ is a disease-specific questionnaire for LSS assessing symptom severity and physical function [30, 31]. The symptom severity scale ranges from 1.0 to 5.0. The physical function scale ranges from 1.0 to 4.0. For both scales, 1.0 is minimum burden. The NRS for leg and back pain ranges from 0 (no pain) to 10 (worst pain imaginable) [10].

The primary outcome measure was defined as a reduction of at least 30% of the ODI score after the 2-year follow-up period, determined as a threshold value to define the surgical intervention as a success [3, 4, 8, 28].

Secondary patient-reported outcomes were summary scores reported at 2-year follow-up for ODI, ZCQ, and NRS for leg and back pain.

### Statistics

We conducted an analysis of data collected prospectively in a RCT, nested within the NORDSTEN-SST. Paired-sample *t*-tests were utilized to compare differences in means at baseline and the 2-year follow-up. To analyze the association between MRI findings and outcomes, multivariable regression models including MRI parameters and controlling for relevant patient demographics, i.e., age (continuous), sex, smoking status (yes/no), and BMI (continuous), were applied. Pfirrmann score, Schizas grade, and DSCA were used as covariates. For the primary dichotomous outcome, a logistic regression model estimating odds ratios and corresponding 95% confidence intervals was applied. For the continuous secondary outcomes, we used linear regression analysis and estimated unstandardized regression coefficients with corresponding 95% confidence intervals. As the proportion of missing information was low, complete case analyses were done. Analyses were performed using Stata (version 16.1).

## Results

### Baseline data

This study included 230 men and 207 women with mean age 66.8 (SD 8.3), in total 437 patients. However, only 414 of the patients had MRIs with adequate visualization of the foraminal canal. The number of patients not returning complete PROMS at follow-up was 46 (11%) including 29 (7%) patients with total dropout. The distribution of index levels was as follows: 24 patients at L2–L3, 146 patients at L3–L4, and 245 patients at L4–L5. The part of the cohort labeled with Lee grades 2–3 presented a higher portion of females (67% vs 47%), more smokers (25% vs 21%), and less patients with higher education (0% vs 29%) when compared to the patients labeled with Lee grades 0–1. Patient characteristics are presented in Table 2.

**Table 2:** Baseline data. Key parameters of the NORDSTEN-SST cohort

	Lee 0–1	Lee 2–3	Cohort in total
Age mean (SD)	66.7 (8.3)	65.8 (11.2)	66.8 (8.3)
Male gender %	53.4	33.3	52.9
Smoker %	20.8	25	20.8
BMI mean (SD)	27.7 (4.2)	26.8 (3.6)	27.8 (4.2)
Higher education %	29	0	28.5

### Radiological findings at baseline

**Fig. 2:**
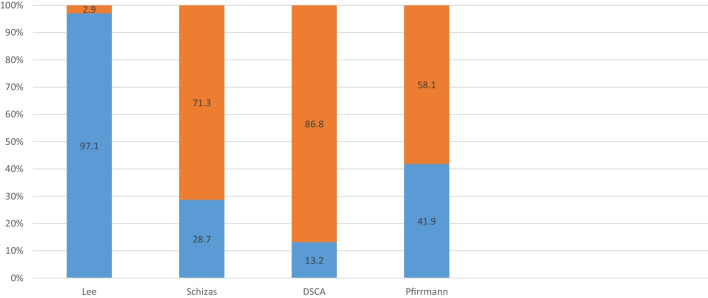
Baseline data. Illustration of the percentage of patients with worst dichotomy score (orange) in each radiological classification system

The cohort presented a high degree of degenerative changes preoperatively. The percentage of patients with severe scores were as follows: Lee (2–3) 2.9%, Schizas (C–D) 71.3%, cross-sectional area (< 75mm^2^) 86.8%, Pfirrmann (4–5) 58.1%. Results are presented in Fig. 2.

The grade of foraminal stenosis on the index level based on Lee’s classification was as follows:

- Lee score 0: 347 patients
- Lee score 1: 55 patients
- Lee score 2: 11 patients
- Lee score 3: 1 patient

### Improvement after surgery

Of the patients with none to moderate foraminal stenosis, 264 of 374 (71%) achieved a minimum of 30% improvement in ODI. Among the patients with severe foraminal stenosis, 4 of 11 (36%) achieved a minimum of 30% improvement in ODI.

### Associations between MRI findings of foraminal stenosis and PROMs

For the primary outcome, a significant association between severe foraminal stenosis on MRI and less chance of reaching 30% improvement in ODI score with OR 0.22 (95% CI 0.06, 0.83; *p*=0.03) was detected. The logistic regression model is given in Table 3.

**Table 3:** Lumbar spinal stenosis patients treated by microsurgical decompression surgery. Logistic regression model with odds ratio indicating the chance of successful surgery when comparing moderate/severe changes in given radiological classification systems. Successful surgery determined as at least 30% improvement in the ODI score from baseline to 2 years post-operative. Adjusted for sex, age, smoking status, and BMI

Variable	Odds ratio	*p*-value	95% CI	*n*
Lee				
0–1 (*n*=402) vs	1			
2–3 (*n*=12)	0.22	0.03	0.06, 0.83	414
Schizas				
A–B (*n*=119) vs	1			
C–D (*n*=296)	1.43	0.22	0.81, 2.55	415
DCSA				
≥ 75 mm^2^ (*n*=55) vs	1			
< 75 mm^2^ (*n*=360)	1.33	0.46	0.62, 2.84	415
Pfirrmann				
1–3 (*n*=174) vs	1			
4–5 (*n*=241)	0.53	0.01	0.32, 0.86	415
Male	1			
Female	0.64	0.07	0.40, 1.03	
Age	0.99	0.41	0.96, 1.02	
Smoker				
No	1			
Yes	0.38	0.00	0.21, 0.69	
BMI	0.93	0.02	0.88, 0.99	

In the secondary analysis when investigating associations between MRI findings and continuous PROMs, a significant association between severe foraminal stenosis and less improvement of ODI with a mean difference of 9.28 ODI points (95%CI 0.47, 18.09; *p*=0.04) was detected. The analysis also showed a significant association between severe foraminal stenosis and NRS back pain with a mean difference of 1.89 (95% CI 0.30, 3.49; *p*=0.02). No association was detected between severe foraminal stenosis and NRS leg pain, ZCQ function, or ZCQ symptoms. The results are presented in Table 4.

**Table 4:** Cohort of LSS patients selected for surgical treatment. Multivariable linear regression model investigating the association between preoperative radiological parameters and improvement in disability/pain scores after surgery. Radiological parameters dichotomized in categories for moderate and severe degenerative change. Severe change analyzed with moderate change used as reference. Given as coefficients (gradients) with CI and *p*-value. All PROMs analyzed as continuous variables controlled for baseline values

	Instrument				
Variable	ZCQ symptoms	ZCQ function	NRS pain in lower extremity	NRS pain in lumbar region	ODI
Lee					
0–1 (*n*=402) vs 2–3 (*n*=12)	0.41 (− 0.07, 0.88) *p*=0.09	0.19 (−0.16, 0.55) *p*=0.29	1.14 (−0.51, 2.78) *p*=0.18	1.89 (0.30, 3.49) *p*=0.02	9.28 (0.47, 18.09) *p*=0.04
Schizas					
A–B (*n*=119) vs C–D (*n*=296)	−0.15 (−0.35, 0.05) *p*=0.15	−0.13 (−0.28, 0.02) *p*=0.10	−0.71 (−1.42, −0.01) *p*=0.05	−0.23 (−0.91, 0.46) *p*=0.52	−4.79 (−8.53, −1.06) *p*=0.01
DCSA					
≥ 75 mm^2^ (*n*=55) vs < 75 mm^2^ (*n*=360)	0.00 (−0.27, 0.27) *p*=1.00	−0.10 (−0.30, 0.10) *p*=0.34	0.28 (−0.67, 1.23) *p*=0.57	−0.49 (−1.40, 0.42) *p*=0.29	0.45 (−4.52, 5.43) *p*=0.86
Pfirrmann					
1–3 (*n*=174) vs 4–5 (*n*=241)	0.15 (−0.01, 0.31) *p*=0.07	0.12 (−0.00, 0.24) *p*=0.05	0.45 (−0.12, 1.03) *p*=0.12	0.35 (−0.20, 0.90) *p*=0.22	2.56 (−0.50, 5.61) *p*=0.10

Unadjusted scores for PROMs at baseline and at follow-up for cohort in total and dichotomized in Lee score none to moderate/severe are demonstrated in Tables 5 and 6.

**Table 5:** Total cohort of LSS patients selected for surgical treatment. Cohort with PROM scores at baseline and 2-year follow-up with *p*-values indicating significant difference

Baseline		2-year follow-up	*p*-values
ODI mean (SD)	38.4 (14.6)	18.8 (16.4)	*p*<0.01
ZCQ mean			
Sympt. (SD)	3.4 (0.6)	2.3 (0.9)	*p*<0.01
Function (SD)	2.5 (0.5)	1.7 (0.7)	*p*<0.01
NRS mean			
Leg (SD)	6.5 (2.0)	3.0 (2.9)	*p*<0.01
Back (SD)	6.3 (2.2)	3.6 (2.9)	*p*<0.01

**Table 6:** Mean values of unadjusted PROMs divided into moderate or severe Lee score with *p*-values based on paired-sample *t*-tests

PROMs	Lee 0–1 (*n*=402)	Lee 2–3 (*n*=12)	
*At baseline*			
ODI (SD) *n*=407	38.4 (14.4)	42.4 (18.4)	*p*=0.35
ZCS function (SD) *n*=406	2.5 (0.5)	2.6 (0.6)	*p*=0.74
ZCS symptom (SD) *n*=403	3.4 (0.6)	3.4 (0.7)	*p*=0.78
NRS leg (SD) *n*=391	6.4 (2.0)	6.7 (2.5)	*p*=0.73
NRS back (SD) *n*=394	6.3 (2.2)	6.8 (2.1)	*p*=0.48
*At 2-year follow-up*			
ODI (SD) *n*=385	18.5 (16.4)	29.5 (15.9)	*p*=0.03
ZCS function (SD) *n*=382	1.7 (0.7)	1.9 (0.7)	*p*=0.32
ZCS symptom (SD) *n*=381	2.3 (0.9)	2.7 (0.8)	*p*=0.16
NRS leg (SD) *n*=369	3.0 (2.9)	4.2 (2.5)	*p*=0.16
NRS back (SD) *n*=372	3.6 (2.9)	5.6 (1.5)	*p*=0.02

## Discussion

The main observation in this study was the association between severe foraminal stenosis of the lumbar foramina and significantly reduced chance of reaching a 30% improvement in the ODI score after microsurgical decompression for lumbar spinal stenosis. Severe foraminal stenosis at baseline reduced the probability to reach the targeted improvement by nearly fivefold when compared to having none to moderate foraminal stenosis. Significant association was also detected between severe foraminal stenosis and NRS back pain but not with NRS leg pain.

The results are in accordance with considerations by Burton in 1981. Burton et al. emphasized that narrowing of the foraminal canal is an integral part of the degenerative process that leads to lumbar spinal stenosis. The group also postulated that nerve entrapment in the foraminal canal is an symptom aggressor [6].

Regarding the prevalence of foraminal stenosis in patients referred to surgery for LSS, the earlier published data contains inhomogeneous study cohorts [16]. It is well known that foraminal stenosis is linked to olisthesis, i.e., slipping of the vertebrae with consequently less space for the nerve in the foraminal canal [18]. Kunogi et al. reported a foraminal stenosis rate preoperatively of 8% in patients with degenerative lumbar disease [23]. To the authors’ knowledge, no earlier studies with similar aim have excluded the patients with olistheses. Therefore, our rate of 2.9% with Lee grade 2 or 3 seems plausible. However, a study cohort with foraminal stenosis listed as an inclusion criterion would likely returned a higher prevalence. Among the candidates for the NORDSTEN-SST trial, a number of patients with LSS and additional foraminal stenosis might have been guided out of the study before inclusion.

Although the aspect of foraminal stenosis as an integrated part of the lumbar spinal stenosis entity is documented [20], there is limited published knowledge regarding the clinical consequence. Yamada et al. using the JOA score and a VAS scale detected a higher prevalence of leg pain at rest in patients with lumbar foraminal stenosis compared with patients with predominantly central lumbar stenosis [33]. In a study by the NORDSTEN group, disk degeneration was found to be a predictor for less postoperative improvement in the ODI score [7]. The NORDSTEN group has formerly investigated potential associations between improvement in PROM and a range of MRI findings but could not detect any associations with likely clinical relevance when investigating Schizas score, DSCA, tropism, or amount of fatty infiltration of multifidus muscle [7].

A puzzling finding in the present study is the association between severe foraminal stenosis and outcome measured with NRS back pain while no such association was detected between severe foraminal stenosis and outcome measured with NRS leg pain. Published spinal literature suggests that decompression of the spinal nerve mainly helps radiating discomfort in patients with LSS [21], although several earlier studies have suggested that lumbar spinal decompression is also beneficial for back pain in LSS patients [19, 22]. The traditional clinical perception is that foraminal stenosis mainly induces leg pain [27]. As a consequence, the presumption is that surgery for LSS with foraminal stenosis is mostly beneficial for leg pain. May foraminal stenosis as a part of central lumbar spinal stenosis have other clinical properties than isolated foraminal stenosis? This is a potential topic for future studies.

### Limitations

The major limitation of this paper is the low number of patients with severe foraminal stenosis in the investigated cohort. Consequently, a warning is in place not to consider our findings as clinical guidelines. Foraminal stenosis was not an inclusion nor exclusion criterion in the NORDSTEN-SST study but an additional radiological finding in a cohort with symptoms of predominantly central LSS. In the present study, we wanted to investigate the treatment of such patients. The question is whether a practice of posterior microsurgical decompression is beneficial for this group. The clinical syndrome of LSS is associated with comorbidity and high age. The practice of performing a simple posterior microsurgical decompression instead of the use of interbody devices, foraminotomy, or far lateral approaches is often tempting but there is a high degree of uncertainty regarding the influence on outcome. We hope that this paper is a start of further studies in this field. Only future investigations will show if the arguments we put forward are legit.

We cannot rule out the possibility that patients with severe foraminal stenosis may be associated with more degeneration of the spine and consequently will improve less after surgery, i.e., foraminal stenosis might be confounded by general lumbar degeneration. The analysis was adjusted for several MRI findings but the absence of other markers for degeneration (e.g., facet joint arthrosis) in the analyses might be a source of bias. This might also explain the detection of significant association between severe foraminal stenosis and ODI/NRS back pain while no such association was suggested between foraminal stenosis and NRS leg pain.

The MRIs investigated in the present study are acquired in supine position. The size and formation of the neuro-foramina are affected by the weight bearing and load [13]. Consequently, patients might have a higher degree of stenosis than observed at point of imaging. When investigating potential foraminal stenosis, we have used the validated method by Lee with sagittal T1-weighted images. Although partial volume effects are inevitable when imaging nerves at 3–5-mm slice thickness, the investigators did not experience problems differencing between blockade of the perineural fat and partial volume effects.

The investigated cohort are patients with predominantly central LSS and corresponding symptoms. According to the inclusion criteria, patients with one-sided radiculopathy are not included in this study. Consequently, the findings in the present study cannot be generalized to a broader specter of patients with foraminal stenosis but only apply to patients with both LSS and a concurrent foraminal stenosis.

The optimal situation would be to include the total of important covariates in the analysis. Albeit, the NORDSTEN data material does not include parameters such as mental status, grade of eventual minimal scoliosis, grade of eventual minimal listhesis, or timespan of symptoms. The inclusion criteria in the present study do however demand a symptom period of minimum 3 months. The exclusion criteria deny patients with a lumbosacral scoliosis of more than 20 degrees verified on AP view or olisthesis of 3 mm or more. However, the impact of mental status, minimal olisthesis, minimal scoliosis, or other non-assessed covariates might influence the results of our analysis. The lack of data regarding any minimal scoliosis and concurrent coupling movements is a major weakness in our analysis with potential mismeasurements of foraminal stenosis.

The non-adjusted covariate higher education (y/n) is unequally distributed between the groups with none to moderate and severe foraminal stenosis. Consequently, this is a potential confounding factor in our analysis.

Only patients treated with posterior microsurgical decompression were included in this study. The absence of other common decompression techniques, i.e., laminectomy, weakens the external validity of our findings.

#### Conclusion

The presence of severe foraminal stenosis as a part of the LSS entity at baseline suggests less improvement in ODI and NRS back pain after posterior microsurgical decompression.
